# Machine learning to predict distal caries in mandibular second molars associated with impacted third molars

**DOI:** 10.1038/s41598-021-95024-4

**Published:** 2021-07-29

**Authors:** Sung-Hwi Hur, Eun-Young Lee, Min-Kyung Kim, Somi Kim, Ji-Yeon Kang, Jae Seok Lim

**Affiliations:** 1 Department of Oral and Maxillofacial Surgery, Hankook General Hospital, Cheongju, South Korea; 2 Department of Oral and Maxillofacial Surgery, College of Medicine and Medical Research Institute Chungbuk, National University, Chungdae-ro 1, Seowon-Gu, Cheongju, Chungbuk 28644 South Korea; 3 Department of Oral and Maxillofacial Surgery, Chungbuk National University Hospital, 776, 1Sunhwan-ro, Seowon-gu, Cheongju, Chungbuk 28644 South Korea; 4 Department of Anesthesiology and Pain Medicine, Severance Hospital, Yonsei University College of Medicine, Seoul, South Korea; 5 Dental Clinic Center, Chungnam National University Hospital, Sejong, South Korea; 6 Department of Oral and Maxillofacial Surgery, College of Medicine, Chungnam National University, Daejeon, South Korea

**Keywords:** Third molar removal, Risk factors

## Abstract

Impacted mandibular third molars (M3M) are associated with the occurrence of distal caries on the adjacent mandibular second molars (DCM2M). In this study, we aimed to develop and validate five machine learning (ML) models designed to predict the occurrence of DCM2Ms due to the proximity with M3Ms and determine the relative importance of predictive variables for DCM2Ms that are important for clinical decision making. A total of 2642 mandibular second molars adjacent to M3Ms were analyzed and DCM2Ms were identified in 322 cases (12.2%). The models were trained using logistic regression, random forest, support vector machine, artificial neural network, and extreme gradient boosting ML methods and were subsequently validated using testing datasets. The performance of the ML models was significantly superior to that of single predictors. The area under the receiver operating characteristic curve of the machine learning models ranged from 0.88 to 0.89. Six features (sex, age, contact point at the cementoenamel junction, angulation of M3Ms, Winter's classification, and Pell and Gregory classification) were identified as relevant predictors. These prediction models could be used to detect patients at a high risk of developing DCM2M and ultimately contribute to caries prevention and treatment decision-making for impacted M3Ms.

## Introduction

Mandibular third molars (M3M) have the highest impaction rate of all teeth in the human dentition^1^. Although impacted M3Ms may remain asymptomatic indefinitely, their presence can result in numerous pathologies, including pericoronitis, root resorption and dental caries of in adjacent teeth, and odontogenic cysts and tumors^2^. Previous studies have reported that distal caries in mandibular second molars (DCM2M) is strongly associated with impacted M3Ms^3,4^. The prevention of caries is the most suitable strategy in such cases as the prognosis of mandibular second molars (M2Ms) is very poor once distal caries sets in^4^.

Numerous studies have identified the risk factors associated with DCM2Ms caused by the proximity to impacted M3Ms, such as sex, age, position of the contact point between the M3Ms and M2Ms, and the angulation and level of impaction of the M3Ms^3,5–7^. However, considering the multifactorial nature of the development of DCM2M, a single predictive factor is insufficient to accurately predict its occurrence; various factors need to be considered together as a complex. This perspective highlights the limitations of the traditional approach that analyzed each risk factor separately. Moreover, it warrants the need for a new predictive approach, such as machine learning (ML), which can reflect the simultaneous analysis of various factors and the nonlinearity or innumerable complex interactions of the predictors^8^.

In recent years, there has been a surge in the amount of research entailing the application of ML techniques to medical classifications, including caries prediction^9,10^. To the best of our knowledge, there is a lack of studies that have applied ML to the prediction of DCM2Ms caused by impacted M3Ms. Therefore, our goal in conducing this study was to develop and validate five ML models designed to predict DCM2Ms arising from the proximity to M3Ms to provide guidelines for clinical decision making.

## Methods

All experiments were performed in accordance with the guidelines and regulations approved by the Institutional Review Board (IRB No. 2020-06-003) of Chungbuk National University Hospital and informed consent was obtained from all participants.

### Study population and data collection

This study retrospectively enrolled 1321 patients with bilaterally impacted M3Ms, as observed on panoramic radiography and cone-beam computed tomography at the Department of Oral and Maxillofacial Surgery, Chungbuk National University Hospital, between January and December 2019. We only included patients with bilaterally impacted M3Ms to limit the bias arising from the selection of laterality (e.g., right or left side). A total of 2642 M3Ms from 1321 patients were enrolled. The exclusion criteria were as follows: (1) M3Ms with incomplete root formation or missing adjacent M2Ms, (2) dentoalveolar pathologies, (3) craniofacial anomalies or syndromes, and (4) incomplete medical records. The candidate features for developing the models were selected from a literature-based search of previously reported variables: demographic factors (sex, age), and anatomical factors (laterality, contact point, angulation, Pell and Gregory classification)^3–6^. DCM2Ms were retrospectively diagnosed using radiographic examination reviewed by a single experienced examiner to eliminate inter-examiner variability. To prevent false-positive diagnoses of DCM2Ms, the examiner included only evidently advanced carious lesions extending to the dentin on the orthopantomogram. The examiner excluded obscure lesions on the distal root surface of M2M to prevent the misinterpretation of root resorption as caries. All radiographs of the impacted M3Ms were reviewed by a single examiner to determine the levels of impaction, angulation, and contact point with the M2M, based on previously reported criteria (see Supplementary Fig. S1 online)^11,12^. All examinations were repeated after one month, with blinding of the previous values. The presence of DCM2Ms was designated as a dependent variable. Data analysis was performed from September 2020 to October 2020.

### Machine learning

The prediction pipeline was developed as shown in Supplementary Figure S2 (available online). The pipeline was generated from five ML methods, namely logistic regression (LR), random forest (RF), artificial neural network (ANN), support vector machine (SVM), and extreme gradient boosting (XGB) using the caret package provided in the R statistical software version 3.6.3 and R studio^13–15^. The developed pipeline consisted of random splitting of the input dataset into training (n = 1850; 70% of 2642 samples) and testing (n = 792, 30% of 2642 samples) datasets, while maintaining equal proportions of the class ratios in each split. We developed five final ML models to predict DCM2Ms in the training dataset, by tuning the hyper-parameters using the caret package provided with the R statistical software. We used fivefold cross-validation with 10 repeats to prevent overfitting. The relative feature importance, provided in arbitrary units, was calculated using the Boruta algorithm^16^. The receiver operating characteristic (ROC) curves were plotted using ggplot2^17^, and the area under the ROC curve (AUROC) was obtained to assess the model’s performance. The optimal threshold was calculated as the point closest to the top-left part of the plot. The AUROCs were compared using the Delong test. The performance metrics, including the accuracy, sensitivity, and specificity were obtained.

### Statistical analysis

Statistical analysis was conducted using the R statistical software version 3.6.3 and R studio^13,14^. The frequency tables were analyzed using Student’s t-test, the χ^2^ test, and Watson–Williams test, as appropriate. The circular mean and circular standard deviation were used to analyze the circular outcomes (e.g., angulation). Spearman's correlation analysis was performed to demonstrate the correlation between two variables. *P* values < 0.05 (two-sided) were considered statistically significant.

## Results

### Baseline characteristics of patients and correlation analysis

**Figure 1:**
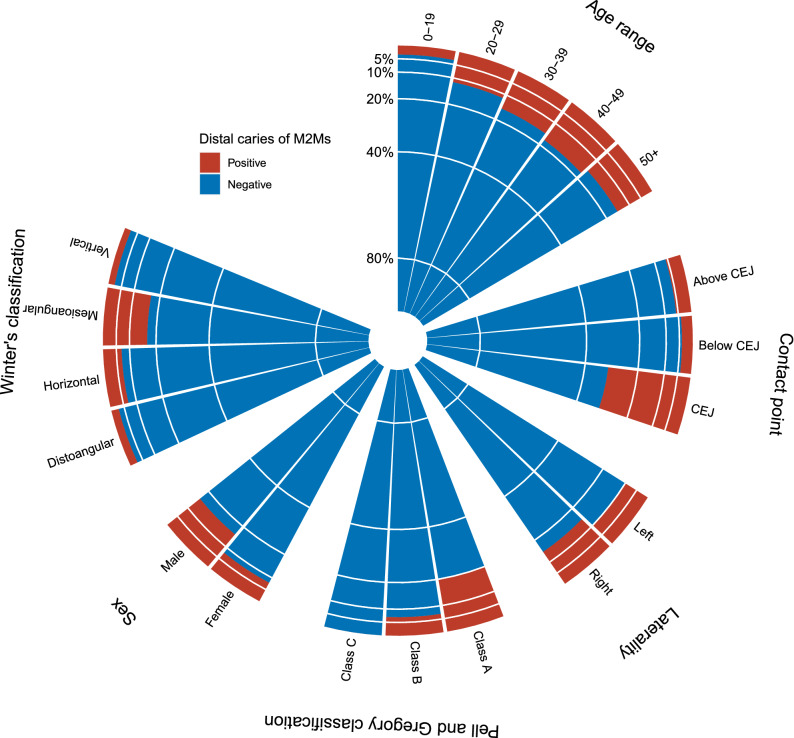
Polar histogram presenting the prevalence of DCM2Ms. *DCM2M* distal caries in mandibular second molars, *M2Ms* mandibular second molars, *CEJ* cementoenamel junction.

The patient's baseline characteristics of patients are depicted in Table 1 and Fig. 1. The proportion of men (70.2% vs. 51.7%, *P* < 0.001), age (30.3 vs. 28.0 years, *P* < 0.001), right-sided involvement (59.9% vs. 48.6%, *P* < 0.001), angulation (53.6° vs. 43.8°, *P* < 0.001), mesioangular impaction (86.6% vs. 60.0%, *P* < 0.001), Pell and Gregory class A (69.6% vs. 38.7%, *P* < 0.001), and contact point at the cementoenamel junction (CEJ) (72.4% vs. 22.1%, *P* < 0.001) was significantly higher in the DCM2M-positive group than that in the DCM2M-negative group. Correlation analysis revealed a slight correlation between the DCM2M-positive group and two variables, namely the contact point (ρ = 0.29, *P* < 0.001), and Pell and Gregory classification (ρ = −0.21, *P* < 0.001) (see Supplementary Fig. S3 online).

**Table 1:** Characteristics of negative (n = 2320) and positive (n = 322) DCM2Ms.

	Negative (%)	Positive (%)	*P*
**Sex**			
Female	1120 (48.3)	96 (29.8)	< 0.001
Male	1200 (51.7)	226 (70.2)	
**Age (years)**			
Mean (SD)	28.0 (9.4)	30.3 (10.2)	< 0.001
**Laterality**			
Left	1192 (51.4)	129 (40.1)	< 0.001
Right	1128 (48.6)	193 (59.9)	
**Angulation**			
Mean (SD)	44.8 (0.63)	54.1 (0.37)	< 0.001
**Winter’s classification**			
Vertical	324 (14.0)	8 (2.5)	< 0.001
Distoangular	231 (10.0)	7 (2.2)	
Horizontal	373 (16.1)	28 (8.7)	
Mesioangular	1392 (60.0)	279 (86.6)	
**Pell and Grogory classification**			
Class A	898 (38.7)	224 (69.6)	< 0.001
Class B	1306 (56.3)	98 (30.4)	
Class C	116 (5.0)	0 (0)	
**Contact point**			
Below CEJ	1136 (49.0)	48 (14.9)	< 0.001
Above CEJ	671 (28.9)	41 (12.7)	
CEJ	513 (22.1)	233 (72.4)	

The circular mean and circular standard deviation were used for the analysis of angulation. *DCM2M* distal caries in mandibular second molars, *CEJ* cementoenamel junction, *SD* standard deviation.

### Development of a prediction model using ML techniques

**Figure 2:**
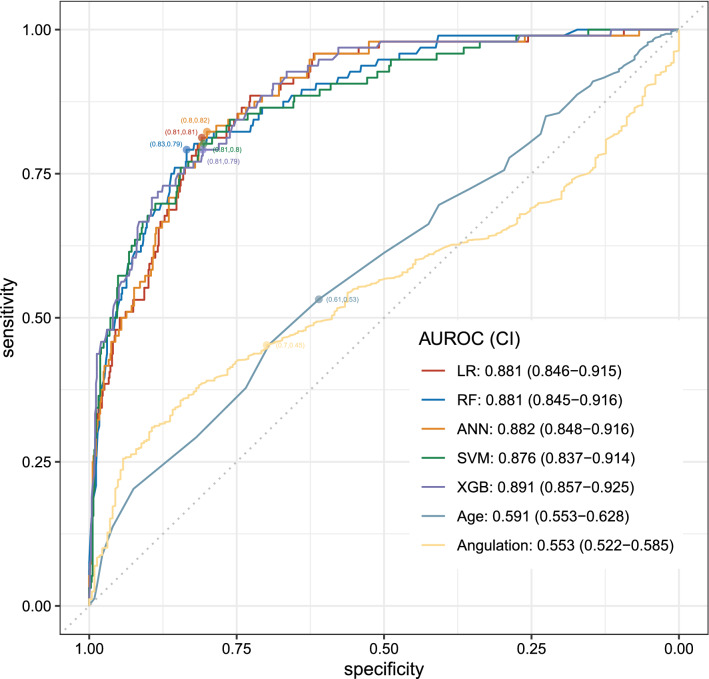
Receiver operating characteristic curves plotted from testing dataset. The optimal threshold is plotted as the point closest to the top-left part of the plot. *AUROC* area under the ROC curve, *CI* confidence interval.

The observed caries ratio was 12.2% (322/2642), which was consistent with the imbalanced data (Table 1). Therefore, we applied the oversampling method to balance the training dataset. We subsequently tested all models using the testing dataset (see Supplementary Fig. S2 online). The AUROCs of all models were > 0.85, indicating that all models performed effectively in the testing dataset. The performance of all ML models was significantly superior to that of single predictors (Fig. 2 and Supplementary Table S1).

### Relative importance of the features

**Figure 3:**
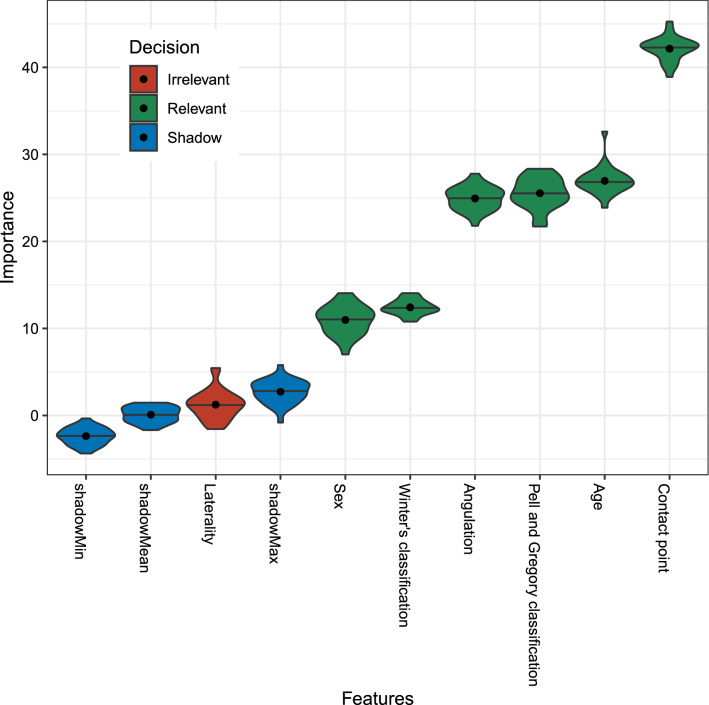
Relative feature importance computed using the Boruta algorithm. The blue violin plots correspond to the minimal, average, and maximum Z scores of a shadow attribute. The red and green violin plots represent the Z scores of the rejected and confirmed attributes, respectively. The black dots and horizontal lines within each violin plot represent the mean and median values, respectively. All features that received a lower relative feature importance than that of the shadow feature were defined as irrelevant for prediction. Laterality was considered as an irrelevant feature (marked in red).

The relative importance of all features was calculated using the Boruta algorithm^16^. One feature, i.e., laterality, was determined as irrelevant for predicting DCM2Ms and the position of the contact point with respect to the CEJ showed the highest relative importance (Fig. 3). The performance of the prediction models, including accuracy, sensitivity, and specificity is shown in Table 2.

**Table 2:** Accuracy, sensitivity and specificity of the prediction models.

Model	Accuracy	Sensitivity	Specificity
LR	0.81	0.81	0.81
RF	0.83	0.79	0.83
ANN	0.80	0.82	0.80
SVM	0.81	0.80	0.81
XGB	0.81	0.79	0.81
Age	0.54	0.53	0.61
Angulation	0.48	0.45	0.70

*LR* logistic regression, *RF* random forest, *ANN* artificial neural network, *SVM* support vector machine, *XGB* extreme gradient boosting.

## Discussion

Herein, we developed ML-based models that were designed to predict DCM2Ms arising due to the proximity to M3Ms, which, to the best our knowledge, has not been attempted before. We also included various performance metrics, including the ROC curve, to enhance the interpretability of the ML models. All five prediction models exhibited comparable accuracy and the value of the AUROC > 0.85 indicated excellent categorization with respect to predictive performance^18^.

Consistent with previous studies^3–6^, our analysis revealed that men, older patients, and patients having mesioangular, horizontal, and Pell and Gregory class A M3M impactions are more likely to develop DCM2M (Fig. 1; Table 1). Moreover, the observed caries ratio (12.2%) is within the range of values reported by previous studies^5,6,19^. With respect to the position of the contact point between M3M and M2M, Toedtling et al. reported that M3Ms with the contact point positioned below the CEJ were most likely to be associated with DCM2Ms^4^. Unlike that study, our analysis and other studies^6^ suggested that the incidence of the contact point at the CEJ was significantly higher in the DCM2M-positive group than that in the DCM2M-negative group. This difference could be attributed in part to our criteria for excluding external root resorption. Despite the diagnostic criteria for the determination of external root resorption on a panoramic radiograph^2^, the radiographic distinction between root resorption and distal caries on M2Ms in proximity to impacted M3Ms is unreliable. In our analysis, patients with an obscure radiolucent lesion on the M2M root surface were excluded, which may have resulted in the exclusion of true carious lesions on the distal root surface of M2Ms, thereby lowering the root caries ratio of M2Ms in proximity to M3Ms with the contact point below the CEJ.

Although patient's baseline characteristics confirmed the risk factors associated with DCM2Ms caused by proximity to impacted M3Ms, a single factor is insufficient to accurately predict DCM2Ms (Table 1 and online Supplementary Fig. S4). As the development of DCM2Ms seems to be simultaneously affected by multiple factors, incorporating combinations of factors and their complex relationships with DCM2Ms to guide treatment decision-making can be challenging for clinicians. In our study, the performance of all ML models was superior to that of single predictors, namely age and angulation, implying that they helped us consider combinations of variables for predicting DCM2Ms (Fig. 2 and online Supplementary Table S1). Interestingly, the combination of a few variables is sufficient to significantly increase the performance of ML models, suggesting that numerous variables are not necessary to generate a good predictive model. In the future, it may be beneficial to compare current models against other ML models employing different combinations of additional features such as oral hygiene and dietary patterns, for predicting DCM2Ms.

In recent years, ML techniques have become increasingly popular tools for analytical healthcare, especially for medical imaging classification^20^. Their recent extensive application can be attributed to the increased availability of electronic health records and advancements in hardware and software^9,21,22^. Despite these advances and the utility of these methods for classification tasks, current ML models still behave as black boxes and fail to provide explanations for their predictions^23^. For example, ML algorithms do not provide information regarding the optimal age for extraction or the onset period for DCM2Ms. Though not providing full interpretability, we have provided the calculated feature importance using the Boruta algorithm, suggesting that age and anatomical factors, such as position of the contact point with respect to the CEJ, angulation, Winter's classification, and Pell and Gregory classification, were determined as relevant for predicting DCM2Ms (Fig. 3). These findings can be interpreted based on the exposure time to plaque. Considering the pathogenesis of dental caries, which is a chronic progressive infectious disease^24^, the duration of exposure to plaque plays a critical role in the development of caries, suggesting that long-standing partially erupted M3Ms steadily increase the caries susceptibility of the adjacent M2Ms. Therefore, the anatomy, i.e., the contact point between the M3Ms and M2Ms and the angulation and impaction level of the M3Ms provide a niche for plaque-accumulation, thereby increasing the exposure time to plaque. In line with this speculation, consistent with our analysis and another study^19^, there were no DCM2Ms in patients with M3Ms classified as Pell and Gregory Class C, which is considered to be completely enclosed by the surrounding bone. However, due to the lack of experimental validation in our study, accurate causal inference for the development of DCM2Ms remains elusive. In the future, prospective microbial experiments investigating plaque on the distal surface of M2Ms are needed to prove and better understand the role of plaque in the development of DCM2Ms.

The limitations of this study should be discussed. First, oral hygiene and diet/sugar intake were not considered in this study. These factors would vary between individuals and populations and be a major contributor to the risk of dental caries development^25^. In the future, it may be beneficial to incorporate these features into ML models in predicting DCM2Ms. Second, the retrospective and cross-sectional nature of this study restricted causal inference. Further prospective studies should investigate the applicability of ML models for the prediction of caries by transforming these retrospective data into a longitudinal research design. Third, our analysis facilitated only speculation regarding the pathogenesis of DCM2Ms with respect to various features, owing to the lack of experimental validation in the ML technique. Recent advances in sequencing technologies and culture-independent methods have better elucidated the associations between the oral microbiome and oral health and disease states, such as dental caries and pericoronitis^26–28^. Further studies using sequencing technologies are needed to understand the microbial changes occurring on the distal surface of M2Ms located adjacent to M3Ms.

DCM2Ms remain a significant concern for clinicians. The anatomical diversity (e.g., C-shaped canals) and low accessibility for instrumentation in M2Ms makes their treatment extremely challenging, thereby requiring expensive and time-consuming restorative treatments, which are often associated with a questionable prognosis^4^. Thus, early detection and prevention of caries is the best treatment option. Hence, our prediction model, which considered various risk factors together as one complex, could be valuable in screening high-risk groups of DCM2Ms caused by proximity to impacted or partially erupted M3Ms.

## Supplementary Information

Supplementary Informations.
