# Supplementary material for: Machine learning to predict distal caries in mandibular second molars associated with impacted third molars
Source: Sci Rep. 2021 Jul 29;11:15447. doi: 10.1038/s41598-021-95024-4 (PMC8322059; doi:10.1038/s41598-021-95024-4)
Supplement: Supplementary file 1 — Supplementary Informations. [file 41598_2021_95024_MOESM1_ESM.pdf]

Supplementary Figure S1. **Representative case of DCM2M**

(a, b) Panoramic and cone-beam computed tomography (axial) showing a carious lesion in the distal surface of the second molar (white arrow). **(c)** The angulation of an impacted third molar was measured based on Winter's classification with reference to the angle formed between the intersected longitudinal axes of the second and third molars. **(d)** Pell and Gregory classification for the occlusal plane.

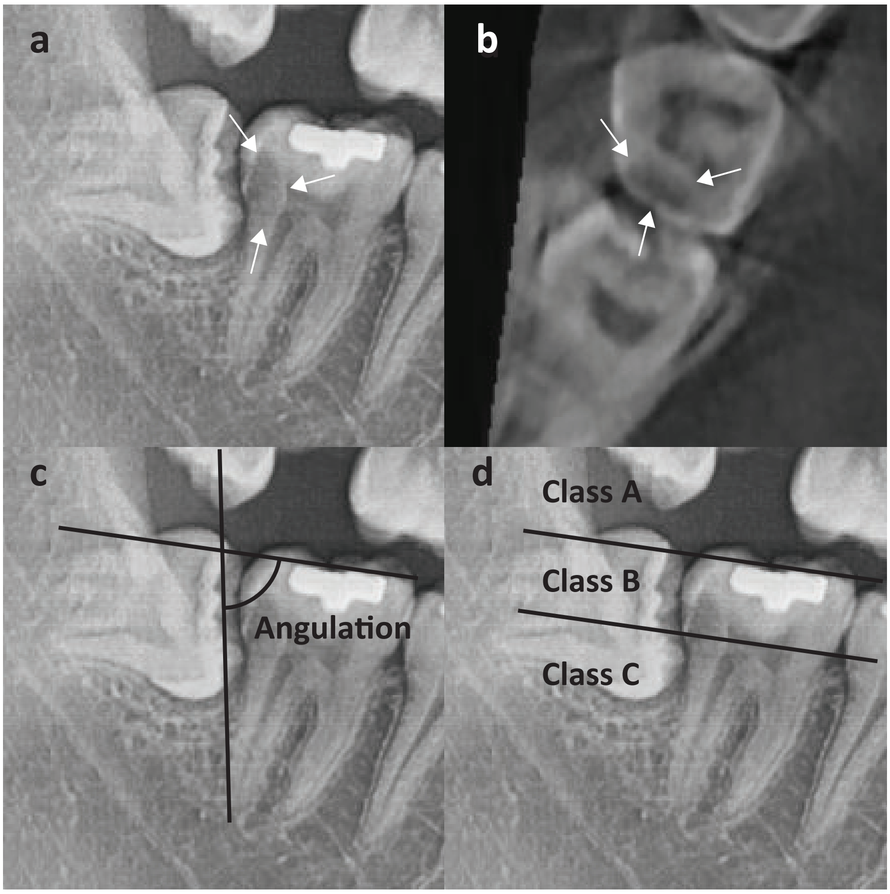

Supplementary Figure S2. **Schematic representation of prediction model development.**

LR: logistic regression, RF: random forest, ANN: artificial neural network, SVM: support vector machine, XGB: extreme gradient boosting

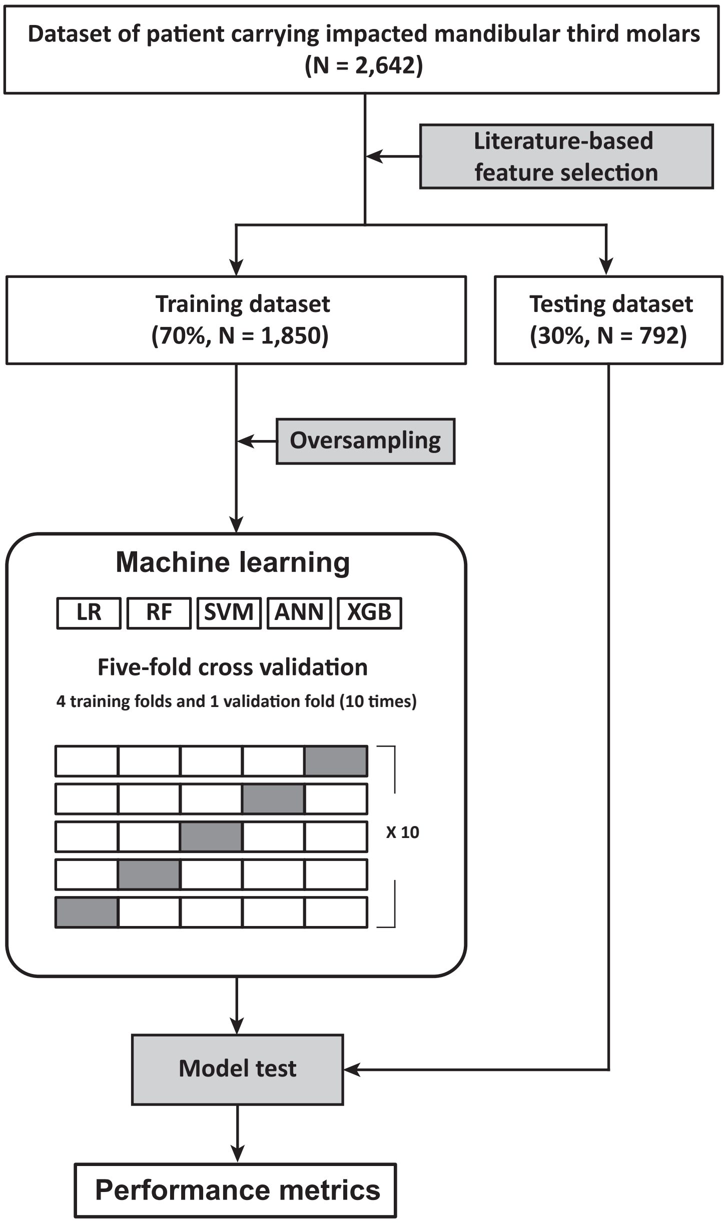

Supplementary Figure S3. Correlation plot of all feature.

The colors represent the degree of pairwise correlation regarding the Spearman's rank correlation coefficient (rho). The darker blue color and larger dot size indicate a stronger positive correlation, whereas the darker red represents a stronger negative correlation. DCM2M: distal caries in mandibular second molars

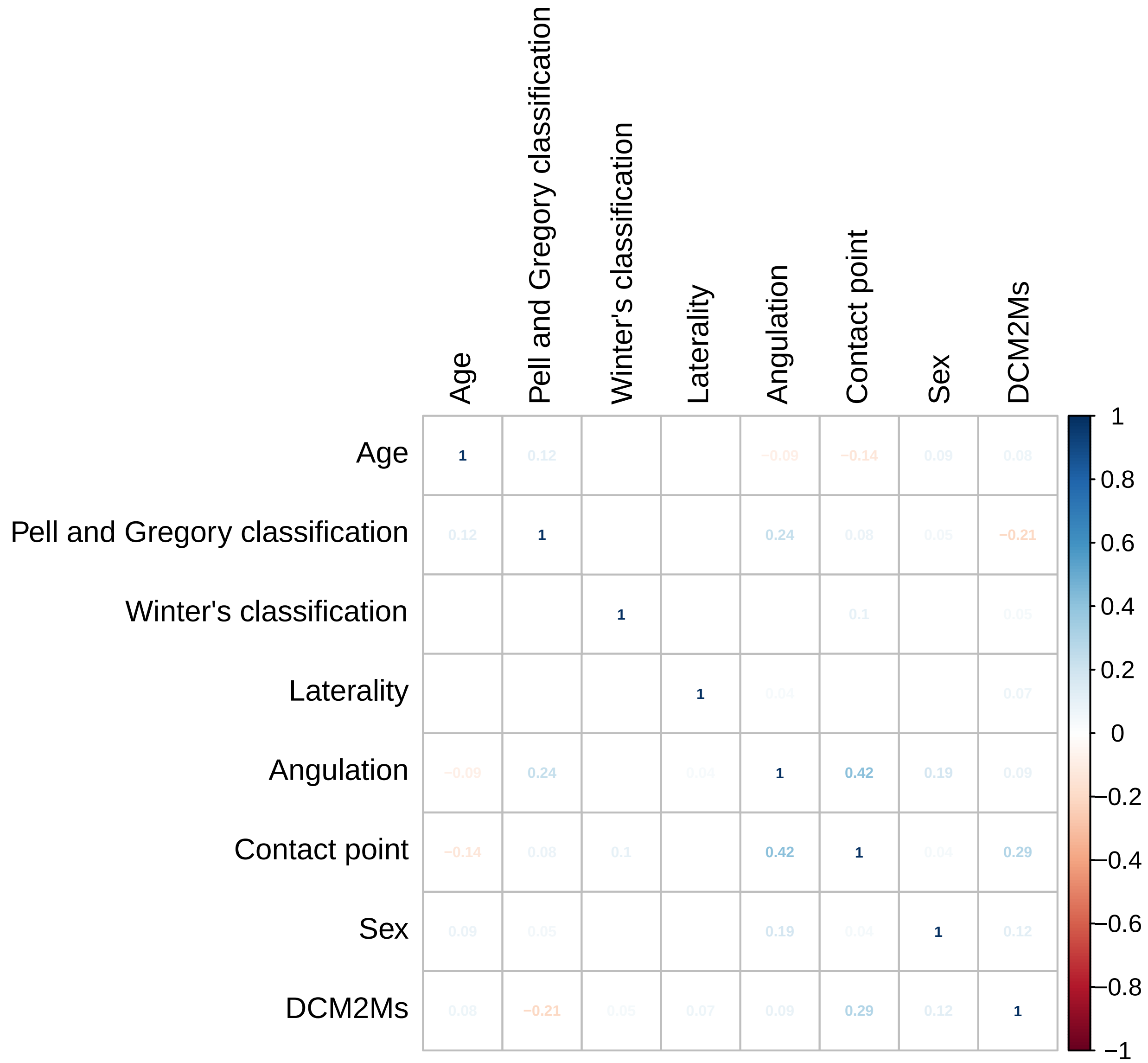

Supplementary Figure S4. Receiver operating characteristic curves plotted from testing dataset using single predictors.

AUROC: area under the ROC curve, CI: confidence interval.

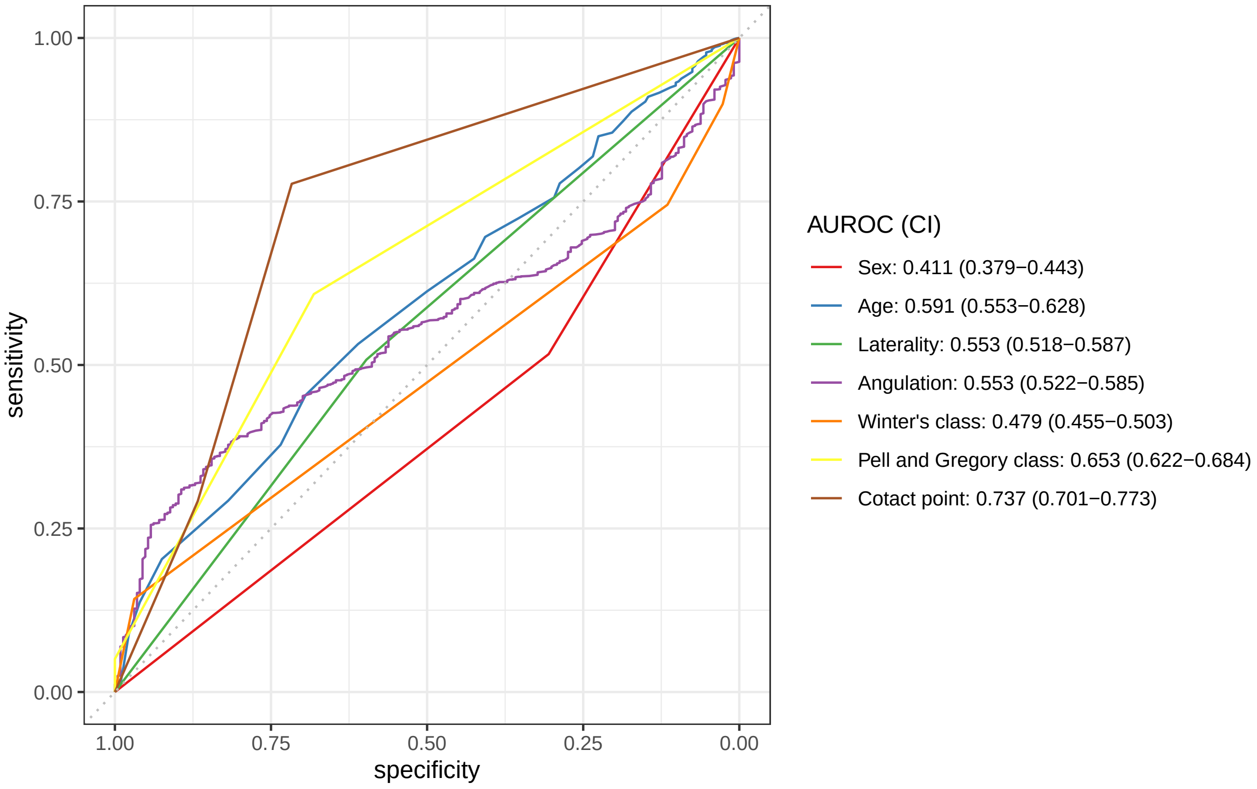

Supplementary Table S1. Calculated probability of the difference between the area under the receiver operating characteristic curve.

DeLong's test was used. *p< 0.001

Model	RF	ANN	SVM	XGB	Age	Angulation
LR	0.506	0.881	0.292	0.957	0.000*	0.000*
RF		0.558	0.309	0.911	0.000*	0.000*
ANN			0.228	0.924	0.000*	0.000*
SVM				0.952	0.000*	0.000*
XGB					0.000*	0.000*
Age						0.082
